# Six- to eight-year-olds’ performance in the Heart and Flower task: Emerging proactive cognitive control

**DOI:** 10.3389/fpsyg.2022.923615

**Published:** 2022-08-11

**Authors:** Claudia M. Roebers

**Affiliations:** Institute of Psychology, University of Bern, Bern, Switzerland

**Keywords:** executive functions, cognitive control, developmental improvements, Heart and Flower task, proactive cognitive control, error monitoring

## Abstract

The Heart and Flower task is used worldwide to measure age-dependent and individual differences in executive functions and/or cognitive control. The task reliably maps age and individual differences and these have consistently been found to be predictive for different aspects of school readiness and academic achievement. The idea has been put forward that there is a developmental shift in how children approach such a task. While 6-year-olds’ tend to adapt their task strategy *ad hoc* and reactively, older children increasingly engage in proactive cognitive control. Proactive cognitive control entails finding the right response speed without risking errors, always dependent on the cognitive conflict. The main goal of the present contribution was to examine children’s adjustments of response speed as a function of age and cognitive conflict by addressing RTs surrounding errors (i.e., errors and post-error trials). Data from a large sample with three age groups was used (*N* = 106 6-year-olds’ with a mean age of 6 years; 3 months; *N* = 108 7-year-olds’ with a mean age of 7 years; 4 months; *N* = 78 8-year-olds’ with a mean age of 8 years; 1 month). Response speed adjustments and the development thereof were targeted both across the Flower and Mixed block, respectively, and within these blocks focusing on errors and post-error slowing. Results revealed evidence for a developmental shift toward more efficient proactive cognitive control between 6 and 8 years of age, with the older but not the younger children strategically slowing down in the Mixed block and smoother post-error slowing. At the same time, we found that even the youngest age group has emerging proactive cognitive control skills at their disposal when addressing post-error slowing in the Flower block. The present study thus tracks the early roots of later efficient executive functions and cognitive control, contributes to a better understanding of how developmental progression in cognitive control is achieved, and highlights new avenues for research in this domain.

## Introduction

The ability to flexibly adjust cognitive processing and behavior for achieving a goal is essential in many everyday life tasks, even for children (e.g., finishing homework rather than playing). A heterogeneous set of regulative cognitive processes is subsumed under the umbrella terms of executive functioning (EF), or more specifically, cognitive control, enabling an individual to inhibit prepotent responses, react flexibly to changing task demands and maintain different task rules or goals in mind (Miyake et al., 2000; Botvinick et al., 2001; Miller and Cohen, 2001; Nigg, 2017). Executive functions are widely studied in children as they have consistently been found to be relevant for academic achievement, health-related behavior, social interactions, and many other developmental outcomes (see meta-analyses: Yeniad et al., 2013; Allan et al., 2014; Jacob and Parkinson, 2015; Follmer, 2018). In this contribution, cognitive control is defined as being a part of the broader term executive function, following definitions of Braver (2012), Chevalier (2015), and Nigg (2017), and acknowledging that in many classical tasks when quantifying sub-components of executive functions cognitive control is also involved. Moreover, by using the term cognitive control rather than executive functions, the selective choice to be made in the task is emphasized, and the intended link to the cognitive control literature is made explicit.

### The Heart and Flower task

The Heart and Flower task plays a significant role in the EF development literature. It is used worldwide, among other reasons, because it can reliably capture individual differences in inhibition and cognitive flexibility across a wide age range (Davidson et al., 2006). The Heart and Flower task consistently documents marked developmental improvements, either concerning accuracy or speed (or both). Researchers typically only include correct responses or RTs for correct responses (Neuenschwander and Blair, 2017; Brod et al., 2019), disregarding errors (and their RTs) as well as trials following errors—although the data would be available. The idea has been put forward that addressing errors and post-error trials may open a new avenue for research on how children gain increasing cognitive control (Lyons and Zelazo, 2011; Roebers, 2017). Error and post-error trials may be informative as they shed light on how children learn to orchestrate the different cognitive processes necessary for mastering both the accuracy and speed demand of this and many other similar tasks. In the present contribution, different aspects of performance in the Heart and Flower task will be targeted, exploring how children achieve the often documented improvements. These will concern children’s accuracy and adaptations of their response speed to maintain a certain level of accuracy: (a) across blocks as the level of cognitive conflict increases, but primarily (b) within blocks, focusing on errors and post-error slowing (slowing of response speed immediately after an error). In other words, blockwise changes in accuracy and speed, as well as trial-by-trial adjustments of response speed within blocks, will be explored as they are discussed as candidate factors for children’s increasing cognitive control (Kail, 1993; Lange-Küttner, 2012; Chevalier, 2015; Kail et al., 2016; Roebers, 2017).

Readers might know the Heart and Flower task: There are three blocks with increasing cognitive conflict. In the first block, hearts are randomly presented on either the right or left side of a screen, and individuals are to press a key on the *same* side where the heart appears. In the second block, flowers appear on either side of the screen, and individuals have to press a key on the *opposite* side where the Flower appears. In the third block, hearts and flowers appear intermixed, and the task is to press the correct key by *applying both rules flexibly*. In every block, the instruction comprises to be as accurate and fast as possible. The Heart block aims to establish a prepotent response; the Flower block is most often used to quantify inhibition; the Mixed block is sought to tap primarily cognitive flexibility.

### Accuracy and speed as dependent measures

There seems to be an implicit agreement among researchers that *accuracy* in the Flower and Mixed block is the most suitable indicator of individual differences in inhibition and cognitive flexibility, respectively, for young children (4- to 7-year-olds’; see, for example, Neuenschwander and Blair, 2017; Stein et al., 2017; Sulik et al., 2018; Rosas et al., 2019; Traverso et al., 2020; Ansari et al., 2021). In contrast, researchers usually use *response times* (RT) on correct trials to map individual differences in older children and adolescents (Davidson et al., 2006; Weintraub et al., 2013; Brod et al., 2019). It is only very recently that researchers have started to question this implicit assumption. One reason for this is the increasing availability of both accuracy and speed (RT) data through computerized testing. Another reason is that there are different suggestions on how to combine accuracy and speed data jointly (e.g., inverse efficient score, balanced integration score, rate correct score, two-vector approaches; see, for example, Bruyer and Brysbaert, 2011; Zelazo et al., 2013; Vandierendonck, 2018; Liesefeld and Janczyk, 2019). However, either of these combined scores has disadvantages and, more importantly, lacks a clear theoretical and empirical basis (Ambrosi et al., 2016; Camerota et al., 2020).

A first step for better understanding the speed and accuracy aspects of performance in children during the transition to school is provided by Camerota and colleagues (Camerota et al., 2019, 2020). In a secondary analysis of the Heart and Flower task used in the Family Life Project (a large sample of 6- to 7-year-olds’), the researchers addressed whether and to what extent accuracy and speed are indicators of a latent EF ability and predictors of academic achievement. They found that accuracy and speed interact in predicting academic outcomes: speed rather than accuracy was the more informative predictor for achievement in highly accurate, but not in average- or low-accurate children. Furthermore, the authors reported evidence for a shift in children’s strategy to master the task: When the cognitive conflict was low (in the Flower block), faster response speed was a better EF indicator than accuracy. In contrast, slower rather than faster responses indicated better EF ability under increasing cognitive conflict (in the Mixed block).

### A developmental shift in cognitive control?

Thus, children seem to approach a cognitive conflict task differentially, depending on the degree of cognitive conflict and their level of accuracy, respectively. In this context, Chevalier and colleagues have proposed a development shift in cognitive control around 5–6 years to explain the above-noted results (Chevalier et al., 2013; Chevalier, 2015). While younger children predominantly engage in reactive control (employed retrospectively after unforeseen events or changes in conflict and when facing unpredictable interference), they increasingly learn to recruit proactive cognitive control resources. Proactive control allows anticipating cognitive conflict, engaging in the ideal amount of mental effort to prevent interference, and finding the best response speed (fast, but not too fast to avoid errors). Proactive control in older children and adults is typically very efficient but also cognitively highly demanding (Wright and Diamond, 2014; Kubota et al., 2020). Using different task paradigms, Chevalier and colleagues have accumulated evidence that developmental progression in cognitive control is—at least in part—due to an increased ability to control proactively (Chevalier et al., 2020; Niebaum et al., 2020). This does not mean that 5- to 6-year-old children cannot engage in proactive control. For example, even 5-year-olds’ responded systematically slower on incongruent than congruent trials in a Flanker, Stroop, or Simon task (Ambrosi et al., 2016), suggesting that there are already early signs of proactive cognitive control (see also Ambrosi et al., 2019).

While these previous studies addressing the above-mentioned developmental shift in cognitive control focused exclusively on children’s *correct* responses under varying cognitive conflict conditions, the present contribution will target *incorrect* responses and responses after incorrect (*post-error*) responses (and their respective RTs). On the one side, incorrect responses are informative as they mirror children’s difficulties in finding a good balance between accuracy and speed. Typically, individuals’ RTs for incorrect responses are substantially slower than for correct responses, with the difference between the two being more pronounced in children than in adults (Gerardi-Caulton, 2000; Jones et al., 2003; Simpson et al., 2012; Danovitch et al., 2019). On the other side, post-error responses tackle an individual’s ability to monitor performance (Wessel, 2012; Chevalier, 2015; Roebers, 2017). Slower post-error compared to post-correct responses are assumed to indicate both reactive cognitive control (allocating control resources toward the committed error) and proactive control (slowing down response time to avoid future mistakes; Botvinick et al., 2001). Post-error slowing thus constitutes an important yet often overlooked aspect for understanding developmental progress in cognitive control (Śmigasiewicz et al., 2020). A developmental shift in cognitive control might consequently consist of less pronounced post-error slowing in older vs. younger children (Brewer and Smith, 1989).

### Post-error slowing

There is not one central theoretical account for post-error slowing. Although it is not the aim of the present contribution to compare different theoretical explanations with each other, these shall nonetheless be briefly mentioned: Post-error slowing (PES) can be attributed to increased cognitive control effort (Botvinick et al., 2001), orienting reactions toward the error (Notebaert et al., 2009), trial-by-trial response threshold adjustments (Danielmeier and Ullsperger, 2011), and increased motor inhibition (Ridderinkhof, 2002). These accounts are considered complementary and vary substantially across different cognitive conflicts (Wessel, 2012). In cognitive neuroscience studies, electrophysiological markers of error processing are typically the error-related negativity (ERN/Ne), a fast response (50–150 ms after a response), and the error positivity (Pe), a positive deflection peaking around 200–500 ms post-error, localized to the anterior cingular cortex (Czernochowski, 2014; Smulders et al., 2016; Schroder et al., 2017; Śmigasiewicz et al., 2020).

A relatively small number of studies have addressed post-error slowing in children (Fairweather, 1978; Brewer and Smith, 1989; Gupta et al., 2009; Smulders et al., 2016; Schroder et al., 2017; Thaqi and Roebers, 2020). Different cognitive conflict tasks and divergent ways to quantify post-error slowing make firm conclusions across studies challenging (Schroder et al., 2019). Nevertheless, studies often document coarser (more exaggerated) trial-by-trial adjustments of response times surrounding errors (overspeeding responses leading to errors and pronounced PES) in younger than older children (Fairweather, 1978; Brewer and Smith, 1989; Smulders et al., 2016; Thaqi and Roebers, 2020).

### The present study

In the present contribution, a detailed behavioral look at 6- to 8-year-olds’ performance on the Heart and Flower task will be presented, exploring how children handle different cognitive conflicts and how they react after committing an error. This is done with the major aim to detect a developmental shift toward increasingly proactive cognitive control (Chevalier, 2015). Thereby, main emphasis will be laid on response speed of error trials and trials following an error. Uncovering how children approach the task, how they react on error trials and trials following an error, and delineating developmental changes in this age range will help better understand the dynamics of cognitive control and how children come to orchestrate the different cognitive processes involved. The aim is to gain detailed knowledge on emerging cognitive control skills and the specific difficulties children have to overcome by addressing primarily errors and sequential effects surrounding errors. Insights into the developmental roots of subprocesses within cognitive control including post-error slowing are helpful for both theoretical advances in EF development and efforts to improve EFs in this critical age range.

The widely used Heart and Flower task has the additional advantage that many other research groups might be interested in and benefit from the results reported below. Records of reaction times for incorrect responses and post-error slowing are available in most data sets but typically and unfortunately not reported. A better understanding of the different performance aspects in a classical EF task may, in the long run, pertain to the factorial structure, the predictive power of the subprocesses involved, and measurement issues, all of which are discussed in the literature (e.g., Willoughby et al., 2016, 2020; Camerota et al., 2018).

We included three adjacent age groups to cover an important age range (6–8 years) during which maturation of the prefrontal cortex, increasing challenges and demands from the environment, and interactions thereof should contribute to developmental progression (e.g., Diamond, 2013). Firstly and aligning with the EF literature, we expected age-related improvements in accuracy and response speed with increasing age. Secondly, we expected lower accuracy and longer reaction times in the more complex blocks (Flower and Mixed block, respectively) than in the Heart block as the cognitive conflict demands increase. In this respect, all three age groups were expected to engage in proactive cognitive control adjustments, but these adjustments’ efficiency should be best in the 8-year-olds’ (less accuracy loss and less slowing down of RTs across blocks; Chevalier, 2015; Ambrosi et al., 2016).

Thirdly, as to incorrect responses as one major focus of the present approach, we expected incorrect responses to be associated with specifically faster response times, with this effect being more pronounced in the younger than the older age groups (interaction between age and trial type), as 6-year-olds’ inhibitory control skills are still less well-developed (Diamond, 2013). Fourthly and most importantly, the ability to specifically slow down after an error (i.e., PES), indicative of error monitoring, was expected to be observable in all three age groups. However, while it was expected that even 6-year-olds’ are able to show specific post-error slowing indicative of emerging error monitoring skills in the relatively easy Flower block, substantially more sophisticated skills were expected in the older compared to the younger participants, especially under unpredictable changes of cognitive conflict (Mixed block).

## Materials and methods

This paper presents secondary analyses of existing data sets generated in the context of two independent studies in which the Heart and Flower task was used to measure individual differences in executive functioning. Integrating these two data sets led to a large sample of adjacent age groups, enabling to address research questions concerning sequential effects surrounding errors widely overlooked in the literature. Both studies were reviewed and approved by the Ethics Committee of the Faculty of Human Sciences of the University of Bern. The children’s parents all had provided their written informed consent to participate in the study. Before the data collection started, children gave their oral consent and were explained that they could abort the testing at any time without giving reasons and without any consequences. However, no child ever did. Data was entirely anonymous.

### Sample

A total of *N* = 290 children (52% female) was included in the analyses. There were *N* = 104 6-year-olds’ (*N* = 54 girls) with a mean age of 6 years; 3 months (*SD* = 3.6 months; min = 70 months, max = 81 months). Data of *N* = 108 7-year-olds’ (54 girls) with a mean age of 7 years; 4 months (*SD* = 3.2 months; min = 82 months; max = 93 months) were used. The sample of 8-year-olds’ consisted of *N* = 78 children (42 girls) with an average age of 8 years; 1 month (*SD* = 2.9 months; min = 94 months; max = 105). There were no age differences between boys and girls in either age group. Children from the two data sets were excluded from the beginning if (a) they did not commit any errors (and thus did not generate post-error data) or (b) committed too many errors (i.e., less than 80% correct in the Heart block, or less than 50% correct in either the Flower or the Mixed block). For children who commit too many errors, we cannot be sure that they understood the task correctly, despite the practice trials before each block. Moreover, if an individual commits too many errors, this suggests that the task is qualitatively different (high working memory load), and post-error slowing is unlikely to be observed (Brewer and Smith, 1989; Notebaert and Verguts, 2011; Wang et al., 2016; Schroder et al., 2019). All analyses reported below include exactly the same sample as described above.

### Procedure

Due to federal regulations during the COVID-19 pandemic, group testing in small groups of students in spacious rooms was conducted. Children were seated in front of a tablet computer (Samsung Galaxy Tab A7^®^ running on Android) and equipped with headphones and two commercially available external response buttons (Buddy Buttons of Ablenet Inc.), which were connected to the tablet with a response box (“Immo-Reaction Response Box” of Immo-Electronics Inc.) recording reaction times with millisecond’s accuracy. The task was programmed on IONIC, an open source for mobile app development. Instructions and feedback during practice trials were audiotaped; intensive piloting guaranteed child-appropriate language, practical handling, and smooth technical implementation.

### Task

An adapted version of the Heart and Flower task (Diamond et al., 2007) was used. For this task, children were to press the external response button either on the same (Heart trials) or the opposite (Flower trials) side of where the stimulus appeared on the screen (response buttons were positioned on the left and right side of the tablet computer’s stand). Trials always started with a cross in the middle of the screen to alert children (300 ms). Stimulus presentation time was 1.2 s. Inter-stimulus intervals were set to 500 ms. The task consists of three blocks, a congruent block (the Heart block; *N* = 24 trials), an incongruent block (the Flower block; *N* = 36 trials), and a Mixed block (a mix of congruent and incongruent trials; *N* = 60 trials, with *N* = 12 incongruent and *N* = 48 congruent trials).

To allow specific analyses of post-error slowing, Heart and Flower trials in the Mixed block appeared in a pseudo-randomized order, with the constraint that an incongruent trial (Flower trial) should always be preceded and followed by at least one congruent (Heart) trial. This version of the task has been successfully used in two large-scale studies with older children and adults (Dubravac et al., 2020, 2022). Based on post-experiment interviews, we are sure that no individual was able to predict the sequence of trials (Readers are reminded that left and right responses to either trial type were also randomized).

Detailed instructions and four practice trials preceded the critical trials. Another four practice trials were completed if a child committed more than two errors during practice. Children were explained: “*I want you to press as fast as possible but also as accurately as possible*!” Thus, neither speed nor accuracy was given stronger emphasis. For each response, its accuracy and reaction time (RT) was recorded. Reaction times faster than 250 ms were set back to 250 ms as they are typically considered as anticipatory responses (Brocki and Tillman, 2014; Wright and Diamond, 2014). If a child did not respond after 4 s, the program automatically proceeded to the subsequent trial and recorded missing values for the response’s accuracy and reaction time. Overall, there were 0.8% missing values in the data.

## Results

### Analyses plan

The first part of this result section will address performance differences between the three age groups and the three task blocks regarding accuracy and response times. A series of analyses of variance will be reported with age as between-subject factor and block as within-subject factor. Reporting a series of ANOVAs (rather than linear regression analyses) and plotting RTs of interest against each other allows to illustrate successes and failure in cognitive control as well to visualize age-dependent and age-general patterns of cognitive control in an easy-to-grasp way. The second part will report on trial-by-trial adjustments of response speed within the Flower and Mixed block. These include response speed of errors and post-error slowing. Partial η^2^- values (Eta Square) will be reported throughout to allow direct comparison of the effect sizes across analyses. Finally, intercorrelations between accuracy, speed, and post-error slowing will be presented as a function of cognitive conflict (task block). Analyses were run with R (R Core Team and contributors worldwide). Data and R code can be obtained from the author.

### Age differences in performance

**FIGURE 1:**
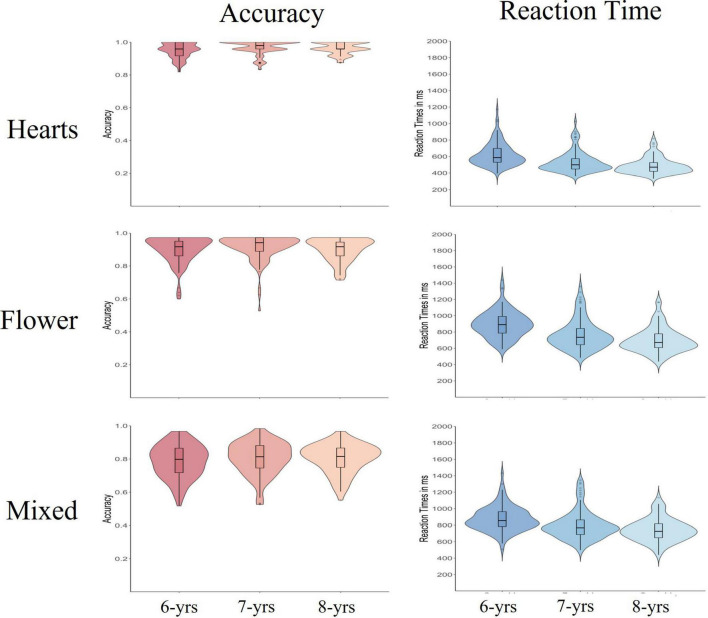
Violin diagrams (combining box and scatter plots) for Accuracy and Reaction Times for the three blocks of the Hearts and Flower Tasks, as a function of age group. The figure depicts the median (horizontal line in the box plot), the lower and upper quartile (borders of the box), 1.5 time the interquartile difference (the whiskers), and the distribution (the external lines).

Figure 1 presents violin diagrams (combining box plots and scatter plots) on the accuracy (left panels) and the reaction times (right panels) as a function of age group and task block.

#### Accuracy

An ANOVA with age group as between-subject factor and block as within-subject factor on children’s accuracy revealed no main effect of age, *F* < 3, n.s., but a strong main effect of block, *F*(2, 574) = 465.65, *p* < 0.001, η*_*p*_*^2^ = 0.62. The interaction between age group and block was non-significant, *F* < 1, n.s. *Post-hoc* tests revealed that, as expected, children reached the highest accuracy in the Heart block (*M* = 0.96, *SD* = 0.04), followed by the Flower block (*M* = 0.90, *SD* = 0.07). Accuracy was lowest in the Mixed block (*M* = 0.80, *SD* = 0.09). As the lack of the interaction between age and block already indicates, the decrease in accuracy across blocks was very similar in the three age groups [6-year-olds’ main effect of block: *F*(2, 206) = 167.78, *p* < 0.001, η*_*p*_*^2^ = 0.62; 7-year-olds’ main effect of block: *F*(2, 214) = 156.04, *p* < 0.001, η*_*p*_*^2^ = 0.59; 8-year-olds’ main effect of block: *F*(2, 154) = 169.34, *p* < 0.001, η*_*p*_*^2^ = 0.69]. In other words, the main effect of task block was very strong, independent of age group.

#### Reaction times

Another ANOVA with age group as between-subject factor and block as within-subject factor was conducted for reaction times. Results revealed strong main effects of age, *F*(2, 287) = 33.27, *p* < 0.001, η*_*p*_*^2^ = 0.19, and block, *F*(2, 574) = 687.49, *p* < 0.001, η*_*p*_*^2^ = 0.71. The effect of block was much stronger compared to the age effect. Averaging across blocks, 6-year-olds’ responded the slowest (*M* = 799 ms, *SD* = 194 ms), followed by the 7-year-olds’ (*M* = 702 ms, *SD* = 200). The 8-year-olds’ responded the fastest (*M* = 644, *SD* = 172 ms). There was also a significant interaction between age group and block for the reaction times, *F*(4, 574) = 3.62, *p* < 0.01, η*_*p*_*^2^ = 0.02. Follow-up analyses on this interaction revealed that the interaction was primarily due to age-specific adaptations between the Flower and the Mixed block, *F*(1, 287) = 5.24, *p* < 0.004, η*_*p*_*^2^ = 0.04: While the 6-year-olds’ did not further decrease their response speed when proceeding from the Flower to the Mixed block, 7-year-olds’ slowed further down. But 8-year-olds’ showed the strongest adaptation from the Flower block to the increased cognitive control demands in the Mixed block. [6-year-olds’ main effect of block: *F* < 2, n.s.; 7-year-olds’ main effect of block, *F*(1, 107) = 4.05, *p* < 0.05, η*_*p*_*^2^ = 0.04; 8-year-olds’ main effect of block, *F*(1, 77) = 10.37, *p* < 0.002, η*_*p*_*^2^ = 0.12]. Comparing the age effects for accuracy and speed, age differences in response speed proved to be stronger compared to accuracy.

### Sequential effects surrounding errors

In this section, sequential effects surrounding errors will be addressed. As accuracy was at ceiling in the Heart block (as intended to establish a prepotent response), this block will not be further considered.

Table 1 shows the number of observations included in the analyses of the Flower block; Table 2 shows the corresponding numbers for the Mixed block.

**TABLE 1:** Number of observations underlying the analyses concerning the Flower block.

Age group	*N*	Correct	Incorrect	Post-correct	Post-error
6-year-olds	104	3,301	256	2,989	256
7-year-olds	108	3,498	239	2,989	239
8-year-olds	78	2,494	212	2,989	212

**TABLE 2:** Number of observations underlying the analyses concerning the different trial types in the Mixed block.

Age group	*N*	Correct	Incorrect	Post-correct congruent	Post-error congruent	Post-correct incongruent	Post-error incongruent
6-year-olds	104	4,778	1,573	2,681	450	489	359
7-year-olds	108	5,120	1,732	2,821	509	583	313
8-year-olds	78	3,701	1,226	2,080	357	387	262

#### Flower block

**FIGURE 2:**
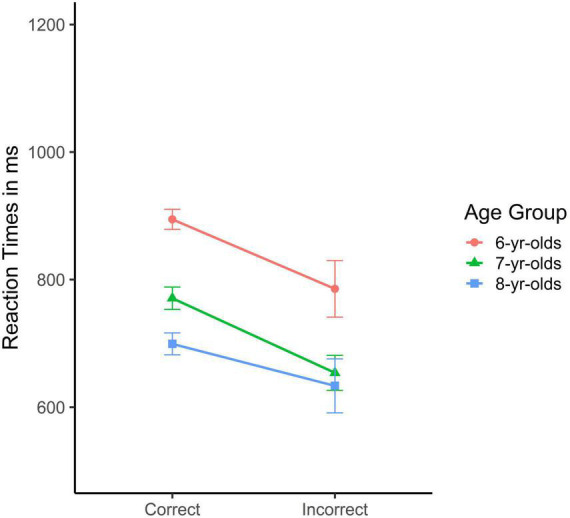
Mean reaction times in msec for the correct and incorrect responses in the Flower block of the Heart and Flower task as a function of age group (error bars represent SEM) showing that participants in all three age group responded faster on incorrect compared to correct trials.

In a first step, children’s reaction times of *correct vs. incorrect trials* were addressed; these are depicted in Figure 2. An ANOVA was conducted on these reaction times with age group as between-subject and correctness of response as within-subject factors. It revealed significant main effects of age, *F*(2, 287) = 14.19, *p* < 0.001, η*_*p*_*^2^ = 0.09, and correctness, *F*(1, 287) = 20.32, *p* < 0.001, η*_*p*_*^2^ = 0.07, but no interaction. As shown in Figure 2, incorrect responses were given at a substantially faster pace than correct responses, independent of age.

**FIGURE 3:**
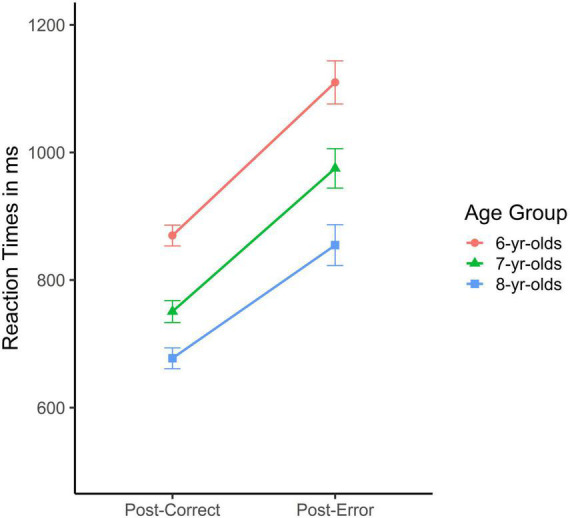
Mean reaction times in msec for correct responses after a correct response (post-correct) vs. after an error (post-error) in the Flower block of the Heart and Flower task as a function of age group (error bars represent SEM) illustrating that children from all three age groups responded slower on post-error trials than on post-correct trials as the cognitive control demands varied strongly from trial to trial.

Next, children’s *post-error slowing* in the Flower block was analyzed by comparing reaction times of the first trial after an error (post-error trial with a correct response) with the first trial after a correct response (post-correct trial with a correct response). These are depicted in Figure 3 (*N* = 6 participants dropped out of these analyses because they had no post-error correct trial but only double errors; one post-error correct trial would have been sufficient to be included in theses and the following analyses; see Schroder et al., 2019). The ANOVA with age group as between-subject factor and trial type as within-subject factor (i.e., post-error vs. post-correct) and reaction times as dependent variable revealed significant main effects of age, *F*(2, 281) = 24.72, *p* < 0.001, η*_*p*_*^2^ = 0.15, and trial type, *F*(1, 284) = 176.46, *p* < 0.001, η*_*p*_*^2^ = 0.39. Thereby, the main effect of trial type was much stronger than the effect of age. The interaction was not significant, *F* < 2, n.s. As can be derived from Figure 3, post-error slowing was present and substantial, independent of age group.

#### Mixed block

When addressing children’s response speed on correct vs. incorrect trials, congruent (Heart) and incongruent (Flower) trials will be analyzed separately.

Although not of primary interest, it is important to establish a congruency effect (considering only correct responses) on the congruent vs. the incongruent trials. There was a significant congruency effect, that is, that there were slower RTs on the incongruent compared to the congruent trials. Independent of age, children responded more slowly on the incongruent compared to the congruent trials [6-year-olds’: congruent: *M* = 820 ms; *SD* = 139 ms; incongruent: *M* = 1,020 ms; *SD* = 256 ms—7-year-olds’: congruent: *M* = 764 ms; *SD* = 158 ms; incongruent: *M* = 959 ms; *SD* = 238 ms—8-year-olds’: congruent: *M* = 702 ms; *SD* = 110 ms; incongruent: *M* = 853 ms; *SD* = 170 ms]. The mixed ANOVA on these correct responses in the Mixed block with age group as between-subject factor and congruency (congruent vs. incongruent) as within-subject factor revealed significant main effects of age, *F*(2, 287) = 13.80, *p* < 0.001, η*_*p*_*^2^ = 0.09, and congruency (congruent vs. incongruent), *F*(1, 281) = 63.60, *p* < 0.001, η*_*p*_*^2^ = 0.18. Thus, it was established that RTs on incongruent compared to congruent trials were slower, independent of age. This documents higher cognitive control demands on the incongruent compared to the congruent trials for all participants.

**FIGURE 4:**
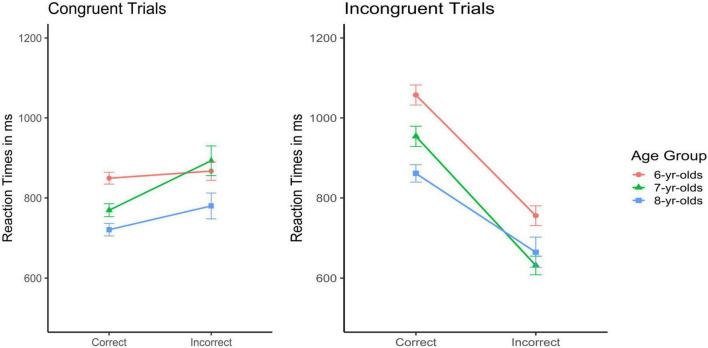
Mean reaction times in msec for correct and incorrect responses in the Mixed Block as a function of trial type (congruent: left panel; incongruent: right panel) and age group (error bars represent SEM) depicting that only on the incongruent trials, incorrect responses were associated with faster RTs.

Turning now to incorrect responses and contrasting those to correct responses, the left panel of Figure 4 shows the mean RT on the *congruent trials* as a function of response correctness and age group (One participant was dropped from these analyses due to missing data). The ANOVA with age as between-subjects factor and response correctness as within-subject factor revealed main effects of age, *F*(2, 286) = 6.49, *p* < 0.001, η*_*p*_*^2^ = 0.04, and response correctness, *F*(1, 286) = 16.93, *p* < 0.001, η*_*p*_*^2^ = 0.06, as well as a significant interaction between age and correctness, F(2, 286) = 4.02, p < 0.02, η*_*p*_*^2^ = 0.03. As shown in Figure 4, incorrect responses to congruent trials in the Mixed block were not given faster than correct responses. On the contrary, in the 7-year olds’, incorrect responses yielded somewhat longer reaction times when there were unpredictable changes in the degree of cognitive conflict. However, this was not the case in the 6- and 8-year-olds’. Readers are reminded here that 8-year-olds’ had generally slowed down more strongly than the 7-year-olds’ in the Mixed block (see above).

On the right side of Figure 4, mean RTs on the *incongruent trials* in the Mixed Block as a function of response correctness and age groups are depicted (*N* = 7 children were dropped from these analyses because of missing observations). As can be seen, when congruent and incongruent trials were intermixed, incorrect responses on the incongruent trials were associated with substantially faster responses compared to correct responses on these trials. The ANOVA with age and response correctness of these incongruent trials in the Mixed block confirmed this impression: There was a main effect of age, *F*(2, 280) = 12.98, *p* < 0.001, η*_*p*_*^2^ = 0.08, a pronounced main effect of response correctness, *F*(1, 280) = 225.03, *p* < 0.001, η*_*p*_*^2^ = 0.45, and a weak but significant interaction, *F*(2, 280) = 4.15, *p* < 0.02, η*_*p*_*^2^ = 0.03. In all three age groups, incorrect responses on the rare incongruent trials were associated with shorter response times, and this effect was somewhat more pronounced in the 7- compared to the 6- and 8-year-olds’. Generally, response speed on these trials was comparable to the response times for the correct responses on the congruent trials (left panel).

**FIGURE 5:**
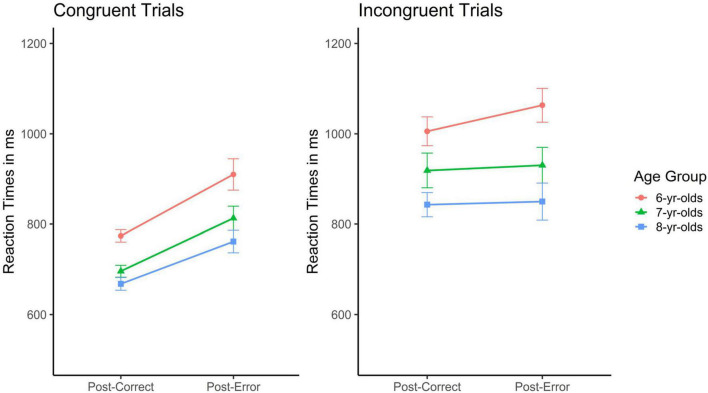
Mean reaction times in msec for post- correct and post-error trials in the Mixed block as a function of trial type (congruent: left panel; incongruent: right panel) and age group (error bars represent SEM) showing that post-error slowing in the Mixed Block showed an inconsistent pattern.

Moving on to *post-error slowing in the Mixed block*, this was first addressed for the congruent trials; results are visualized in the left panel of Figure 5 (*N* = 6 children dropped out of the analyses as they had no errors on the congruent trials with a correct response on the subsequent trial). The ANOVA with age group as between subject and trial type (post-correct vs. post-error) as within-subject factors revealed main effects of age, *F*(2, 281) = 11.40, *p* < 0.001, η*_*p*_*^2^ = 0.08, and trial type, *F*(1, 281) = 54.06, *p* < 0.001, η*_*p*_*^2^ = 0.16, but no interaction, *F* < 1, n.s. Figure 5 illustrates a specific slowing down in participants’ response time following an error. Despite the general age effect with older children responding faster than younger children, the main effect of trial type was stronger than the age effect. Results further revealed that post-error slowing on the congruent trials in the Mixed block was similar across age groups.

Next, *post-error reaction times following errors on an incongruent trial* (in the Mixed block) were addressed. Results are depicted in the right panel of Figure 5. Readers are reminded that a congruent (Heart) trial always followed an incongruent (Flower) trial in the Mixed block. We thus did not expect post-error *slowing* as faster RTs can be expected on the congruent compared to the incongruent trials. Rather, we expected that older compared to younger children would more efficiently adjust their response speed to the change in cognitive conflict, that is, to the lower conflict on the subsequent trial and thus respond faster (*N* = 14 children were excluded from these analyses because they had no post-error correct trials after an error on the infrequent incongruent trials but only double faults). The ANOVA with age group as between subject and trial type (post-correct congruent vs. post-error congruent) as within-subject factors revealed a main effect of age, *F*(2, 273) = 10.07, *p* < 0.001η*_*p*_*^2^ = 0.07, but no main effect of trial type and no interaction, *F*s < 1, n.s. Figure 4 shows that although the post-error trial was congruent and should thus yield *shorter* reaction times, this was *not* the case, independent of age.

### Intercorrelations across the task blocks

In this last section, intercorrelations between the different aspects of Heart and Flower performance will be addressed. Table 3 shows the zero-order correlations between accuracy, RT, and post-error slowing (PES), for each task block separately (Partial correlations controlling for chronological age were also computed; since none of the associations changed substantially, these are not depicted). Inspection of Table 3 reveals that the accuracy, RT, and PES measures intercorrelated substantially across blocks. Thereby, the links between the RTs across blocks are higher (*r* = 0.75–0.62) than the accuracy measures (*r* = 0.23–0.39). Post-error slowing in the Flower block and the Heart trials in the Mixed block also showed a significant association (*r* = 0.28). As to a possible speed-accuracy trade-off, Table 3 reveals that there were no consistent signs of an accuracy-speed trade-off: faster responses were not associated with lower accuracy. Remarkably, there were no significant correlations between PES and accuracy, pointing to the fact that PES does not necessarily lead to higher accuracy, as one might expect, at least not in this young age range.

**TABLE 3:** Zero-order correlations between accuracy, reaction times (RT), and post-error slowing (PES) for the Heart (H), Flower (F), and the Mixed (M) blocks.

Measure	Acc_F_	Acc_M_	RT_H_	RT_F_	RT_M_	PES_F_	PES_*Mcon*_	PES_*Minc*_
Accuracy_*H*_	0.39**	0.23**	–0.02	–0.04	–0.02	0.00	–0.07	–0.09
Accuracy_*F*_	−	0.35**	–0.02	–0.09	–0.02	0.01	–0.12	–0.03
Accuracy_*M*_		−	–0.01	–0.10	0.03	0.09	–0.16	–0.07
Reaction Time_*H*_			−	0.75**	0.63**	0.06	0.08	0.02
Reaction Time_*F*_				−	0.62**	0.09	0.19	0.04
Reaction Time_*M*_					−	0.08	0.22**	0.02
Post-Error Slowing_*F*_						−	0.28**	0.08
Post-Error Slowing_*Mcon*_							−	0.03

Post-Error Slowing_Mcon_,post-error slowing in the mixed block after a congruent (Heart) trial; Post-Error slowing_Minc_, post-error slowing in the mixed block after an incongruent (Flower) trial. ** p < 0.001.

## Discussion

The present contribution provides a detailed look at 6- to 8-year-olds’ performance in the Heart and Flower task. The analyses focused on speed and accuracy across three adjacent age groups, across task blocks and also within task blocks. Thereby, the main focus was laid on response speed of errors and correct responses following an error (post-error slowing) providing an often overlooked perspective on children’s task performance. It was hypothesized that older in comparison to younger children would show less exaggerated adaptations of their response speed following an error (less post-error slowing) pointing to their emerging ability to monitor performance enabling an efficient balance between speed and accuracy.

Concerning developmental differences in either speed or accuracy in the age range of 6–8 years, we found substantial improvements in speed, but no longer in accuracy. One reason for this differential pattern most likely lies in ceiling and close-to-ceiling performance in terms of accuracy in the Heart and Flower block, independent of age, making the detection of strong age effects statistically more difficult. The age effect on RTs explained 19% of the variance, suggesting that RT maps critical age variance very well. This result aligns nicely with the most recent findings reported by Camerota and colleagues (Camerota et al., 2019, 2020), showing that speed may be the better indicator of EF ability even in young children, especially when accuracy is high as in the present sample and these easy task blocks. Of course, this interpretation has to be treated with caution as more complex tasks will likely produce a different pattern of age differences (and different levels of accuracy). In many but not all everyday life situations, speeded and, at the same time, correct responses are advantageous. However, there may be situations where accuracy rather than speed should be emphasized (e.g., dangerous situations in road traffic).

In search for early and emerging roots of later proactive control, all three age groups exhibited the ability to adjust their speed of responses, at least to some degree. For one, independent of age, children slowed down their response speed across task blocks, adjusting to the increasing cognitive conflict in the task. Figure 1 reveals that reaction time increased substantially from block to block (30–50% increases), while accuracy remained relatively high (a decrease of 17–16%). The practice trials before each block informed children about the amount of cognitive conflict, and they adjusted their response speed in a strategic and meaningful manner. The size of the block effect was massive (explaining 71% of the variance in response speed across the blocks). Yet, 6-year-olds’ did not adapt their overall speed of responding between the Flower and Mixed block, while the two older age groups slowed further down, pointing to age-related boundaries of these emerging skills.

For another, when fine-tuned adjustments within blocks were addressed, children of all three age groups were found to specifically slow down after an error, at least to some extent. As hypothesized, even the participating 6-year-olds’ substantially slowed down after an error in the Flower block. In other words, there was substantial post-error slowing in the Flower block, independent of age (Figure 3). There was also evidence for error monitoring in the Mixed block, but only for the less conflicting Heart trials (left panel of Figure 5). To the best of my knowledge, this is the first study to document substantial error monitoring in the Heart and Flower task including children in the age range of 6–8 years. While early signs of error monitoring have repeatedly been documented in the literature on children’s emerging metacognitive skills (Roebers, 2017), addressing error monitoring within executive functioning tasks is still rare. The results on post-error slowing presented here might open new avenues for research aiming to better understand developmental progression in cognitive control. That is, post-error slowing might serve as an additional aspect of task performance. This is because the findings underscore young children’s ability to perceive different degrees of cognitive conflict and act on this perception. Moreover, substantial post-error slowing in this age range confirms the already developed ability to monitor performance but extends this ability to another class of cognitive tasks (Chevalier et al., 2015; Roebers, 2017). The present study thus provides additional evidence on children’s emerging cognitive control skills by uncovering substantial and specific error monitoring skills. It appears that when the cognitive conflict is either predictable (in the Flower block, where all trials are equally conflicting) or not too complex (the Heart trials in the Mixed block), even 6-year-old children can adequately modulate their cognitive control, in this case here by specifically slowing down after an error (Ambrosi et al., 2016, 2019; Chevalier et al., 2020; Niebaum et al., 2020).

There was also evidence for a developmental shift in proactive control (i.e., a significant interaction between age and trial type). Firstly, 7- and 8-year-olds’ slowed down their response speed more strongly between the Flower and the Mixed block than the two younger age groups. They were disproportionately better able to perceive the unpredictable changes in the cognitive conflict in the Mixed block and activate more proactive control resources accordingly allowing them to strategically slow down. This finding is in line with Camerota et al. (2020) reporting that slower (rather than faster) responses in the Mixed block were indicative for better EF skills. Moreover, the two younger age groups made more errors than the 8-year-olds’ on the seemingly easy congruent trials in the Mixed block (see Table 2), underscoring that their cognitive control skills are yet less flexible, less efficient, and less strategic compared to the oldest age group.

Secondly, there was evidence for a developmental shift concerning impulsive errors in the Mixed block. While 6-year-olds’ did no longer modulate their response speed in this most difficult block, 8-year-olds’ remained flexible and adaptive by taking generally more time, and especially for the incongruent than for the congruent trials, 7-year-olds’ were found to “overreact” to the changing task demands by most strongly (and spontaneously) varying their response speed (Figures 4, 5). This pattern of results suggests that specific developmental changes in cognitive control occur in this age range. Eight-year-olds’, but not the younger children, are able to strategically and generally slow down thereby avoiding over-reactions under changing task conditions. They appear to find a better balance between accurate responding (actual trial), error processing (previous trial), stimulus-driven responses, and goal-directed behavior (subsequent trial).

In the most challenging task block, the Mixed block, boundaries of children’s cognitive control skills became obvious. Especially on the incongruent trials, on which always a congruent trial followed (and thus shorter RT could be expected), none of the three age groups showed shorter RTs. This lack of speeding up after an incongruent trial may mirror—at least in part—error monitoring. Following-up on the orienting reaction account (Notebaert et al., 2009) or on the increased response threshold explanation (Ridderinkhof, 2002) of post-error slowing, children appear to allocate cognitive resources toward the committed error impeding their processing of the actual, easy congruent trial. It would be interesting to address sequential effects in the Heart and Flower task in older children as further important developmental improvements with respect to post-error slowing in the Mixed block might be observable.

Increasingly facing challenges and complex cognitive conflicts in everyday life and academic situations that call for children’s developing self-regulatory skills, monitoring performance and perceiving errors might be driving forces for development for cognitive control, but also for other domains of cognitive development. It would be interesting to disentangle the age effects from schooling effects. A cut-off study design may help better estimate the impact of formal schooling on the assumed developmental shift. Future studies may want to address this. A related issue concerns the application of the present findings to instructional contexts. Teachers may want to emphasize that one can learn from mistakes (for example, “*I was working too fast!*”), that mistakes can be productive in many contexts, and that self-monitoring and self-regulated adaptations of cognitive control are beneficial. Providing systematic opportunities for children to make and detect their errors should be part of children’s curriculum early on.

When considering the relations between accuracy, response speed, and post-error slowing as meaningful performance variables of the Heart and Flower task (see Table 3), it appeared that each of these measures is relatively consistent across blocks. Thus, children who tend to be fast in one block tend to be fast in the next block, relatively independent of cognitive conflict. Some authors have suggested that it may be helpful to extract a speed factor when estimating EF ability (Willoughby et al., 2020), and the high correlations found here support this suggestion. Predicting accuracy or post-error slowing from one block to another proved far less accurate. This finding suggests that accuracy and post-error slowing depend more on the kind and amount of cognitive conflict than on response speed. As Wessel (2012) has argued, there is not one single monitoring entity, and monitoring processes are heterogeneous by nature. The low consistency of post-error slowing thus seems to indicate that different error monitoring processes are at work in the different task blocks. This is not surprising as the task blocks have always been considered to target various EF sub-processes. The fact that there was no consistent relation between post-error slowing and accuracy aligns with the literature showing that the relationship is far more complex than had been expected (Wessel, 2012), depending mainly on the kind of conflict (Danielmeier and Ullsperger, 2011; Dubravac et al., 2022), and the efficiency of cognitive control skills (Chevalier et al., 2015; Roebers, 2017).

Some limitations should also be mentioned. For one, there is yet no well-agreed on contrast that best maps post-error slowing (Schroder et al., 2019). For another, issues of reliability have been raised (Schroder et al., 2019). Large sample sizes as in the present case may contact low reliability but yet, the relatively small number of error and post-error trials per child remain a worry. At the same time, however, as a general psychology phenomenon post-error slowing both in children and adults seems to be present in any cognitive control task underlining that it is present, replicable, and worth being investigated (Dubravac et al., 2020, 2022; Thaqi and Roebers, 2020). Moreover, measurement problems associated with reaction times are ubiquitous in behavioral science, not limited to developmental research, but definitely serious (Draheim et al., 2019). Future research needs to tackle these topics, and find alternative or additional ways to best capture task performance in a holistic, multi-dimensional way. My expectation is that error monitoring will be included in such approaches.

Taken together (and despite some measurement issues), the present contribution provided fresh and detailed insights into different performance aspects of 6- to 8-year-olds’ cognitive control. With a distinct emphasis on emerging proactive control, response speed modulations surrounding errors in the different task blocks were addressed. Even 6-year-olds’ were found to engage in proactive cognitive control. However, these skills were fragile, as they still made errors on the easy congruent trials when they were intermixed with incongruent trials. Across and within blocks, there was evidence for the assumed developmental shift toward increased proactive cognitive control in this period in terms of less pronounced post-error slowing in the older compared to the younger children. Improved proactive cognitive control might account for developmental improvements in other subcomponents of executive functions, such as working memory and cognitive flexibility. It may also be an informative process regarding the predictive power of cognitive control for many different developmental outcomes. Further research on trial-by-trial adjustments of response speed and post-error slowing is undoubtedly necessary to uncover the early roots of later efficient self-regulation.

## Data availability statement

The raw data supporting the conclusions of this article will be made available by the authors, without undue reservation.

## Ethics statement

The studies were reviewed and approved by the Ethics Committee of the Faculty of Human Sciences of the University of Bern. The children’s parents all had provided their written informed consent to participate in the study. Children gave oral consent and were informed that they could terminate the task at any time. No child ever did.

## Author contributions

CR is the only contributor of this work, solely responsible, ran the analyses, wrote the manuscript, and revised the manuscript.
