# Erratum: Six- to eight-year-olds' performance in the Heart and Flower task: Emerging proactive cognitive control

**DOI:** 10.3389/fpsyg.2023.1180629

**Published:** 2023-03-15

**Authors:** Frontiers Production Office

**Affiliations:** Frontiers Media SA, Lausanne, Switzerland

**Keywords:** executive functions, cognitive control, developmental improvements, Heart and Flower task, proactive cognitive control, error monitoring

An omission to the funding section of the original article was made in error. The following sentence has been added: “Open access funding was provided by the University of Bern.”

The original article has been updated.

